# Effect of Immunonutrition on the Liver Function Status of End-Stage Liver Disease Patients Waiting/Referred for Liver Transplant: A Randomized Controlled Trial

**DOI:** 10.7759/cureus.36923

**Published:** 2023-03-30

**Authors:** Safeena Beevi SS, Biju Pottakkat

**Affiliations:** 1 Surgical Gastroenterology, Jawaharlal Institute of Postgraduate Medical Education and Research, Puducherry, IND

**Keywords:** end-stage liver disease, supervised diet advice, liver function, malnutrition, liver transplantation, immunonutrition

## Abstract

Background

Malnutrition and liver dysfunction are commonly observed in patients with chronic liver disease. With the current surge in liver diseases, prompt nutritional planning will ensure the well-being of patients during the waiting period and will improve the clinical outcomes following liver transplantation (LT). We conducted this study to monitor the effect of immunonutrition on liver function parameters among end-stage liver disease (ESLD) patients referred for LT.

Methodology

In this randomized controlled trial, 144 ESLD patients who met the inclusion criteria were randomly enrolled into control (CON) and intervention (INT) groups after obtaining informed consent. Three patients were lost to follow-up due to the COVID-19 lockdown. The INT group (n = 69) was provided with 100 g of immunonutrient and the CON group (n = 72) was provided with supervised diet advice. Liver function test (LFT) parameters such as total protein, albumin, total bilirubin, direct bilirubin, aspartate aminotransferase, alanine aminotransferase, alkaline phosphatase, gamma-glutamyl transferase, and prothrombin time/international normalized ratio before and after therapy at one month were checked in both groups.

Results

The majority of patients with the disease were males (83.3% in the CON group vs 76.8% in the INT group), having alcoholism as the etiology in both groups with 45.8% in the CON group and 56.5% in the INT group. The comparison of LFT parameters among ESLD patients during pre and post-therapy between the control and immunonutrition groups did not show any statistically significant difference in the LFT parameters between the INT and CON groups both at baseline and at one month.

Conclusions

The impact of immunonutrition on ESLD patients awaiting LT compared to supervised diet advice did not significantly improve liver function. The liver disease itself profoundly affects the level of nutrition; hence, nutritional assessment and early nutritional interventions can be instituted to improve clinical outcomes.

## Introduction

Chronic liver disease (CLD) is now evolving as a significant public health issue in India. The primary reasons for the increasing trend in CLD are non-alcoholic fatty liver disease (NAFLD) and alcoholic liver disease (ALD) in India due to societal customs and altered lifestyle patterns. Therefore, prompt dietetic intervention among CLD patients is necessary to fight against malnutrition and related complications [1]. Nutrition is vital to patient management, and protein-calorie malnutrition is higher among liver disease patients waiting for liver transplantation (LT). Nutritional assessment in the pre-liver transplant phase aids to plan a better diet plan for these patients [2]. The suitable assessment of patients with cirrhosis should be very robust, and expertise in dietary status will assist to fight incorrect nutrient loss and stabilize the patient’s nutritional level [3].

LT has transformed the management of CLD. Enough intake of protein and calories should be ensured for undernourished cirrhotic patients through diet, oral nutritional supplements, or enteral or parenteral nutrition. Malnutrition further compromises hepatic function. However, trials that support the effectiveness of nutrient supplementation in augmenting patient outcomes after LT are still limited [4]. Studies related to liver function after immunonutrient therapy in CLD patients are also lacking.

## Materials and methods

This randomized controlled trial (RCT) was registered in Clinical Trials Registry-India (CTRI/2019/08/020973). A total of 144 end-stage liver disease (ESLD) patients who attended the Gastroenterology (Medical and Surgical) Department of Jawaharlal Institute of Postgraduate Medical Education and Research, Puducherry were enrolled in this study after obtaining the Institutional Ethical Committee (Human Studies) approval (approval number: JIP/IEC/2018/502). Written informed consent was obtained from all participants. Three patients were lost to follow-up due to the COVID-19 lockdown. The sample size was estimated using the statistical formula for comparing two independent means. The minimum expected difference in the biochemical parameters between the study groups as an impact of the intervention was 10% of the baseline values. The sample size was estimated for the biochemical parameters, and the effect size yielded higher sample size was considered. The sample size was estimated at a 5% level of significance and 80% power. The estimated sample size was 64 in each group, which was further increased to 72 in each group with an expected dropout of 10%. Computer-generated random number sequence with block randomization of varying sizes was generated to randomize the patients. The sequence was generated by a staff in the liver clinic who was not a part of the study. The random sequence was concealed before allocation by the SNOSE technique (serially numbered opaque sealed envelope). The principal investigator allocated the participants to the corresponding arm. Because of the nature of the intervention (difference in the intervention in both arms), blinding the investigator and the participant was impossible. The outcome assessor was also not blinded in this study.

Immunonutrient 100 g was provided to the patients to ensure a compliance rate of 75% which was to be taken orally a day for one month for the intervention group (INT) along with a supervised diet and nutritional counseling. To ensure intake compliance, phone calls were made once every four to five days. In this study, 100 g of immunonutrient (Fresubin Onco powder) provided 415 kcal, with 28 g of protein, 11 g of fat, omega-3/omega-6 fatty acids (2 g/0.70 g), L-arginine 425 mg, glutamine 500 mg, and other essential vitamins and minerals. The control group (CON) was provided with supervised diet advice and nutritional counseling with the same calorie and protein intake for one month along with a diet plan. Subsequently, 5-10 mL of blood was drawn for checking biochemical and liver function parameters at enrolment and at one month for both groups during the clinic visit.

**Figure 1:**
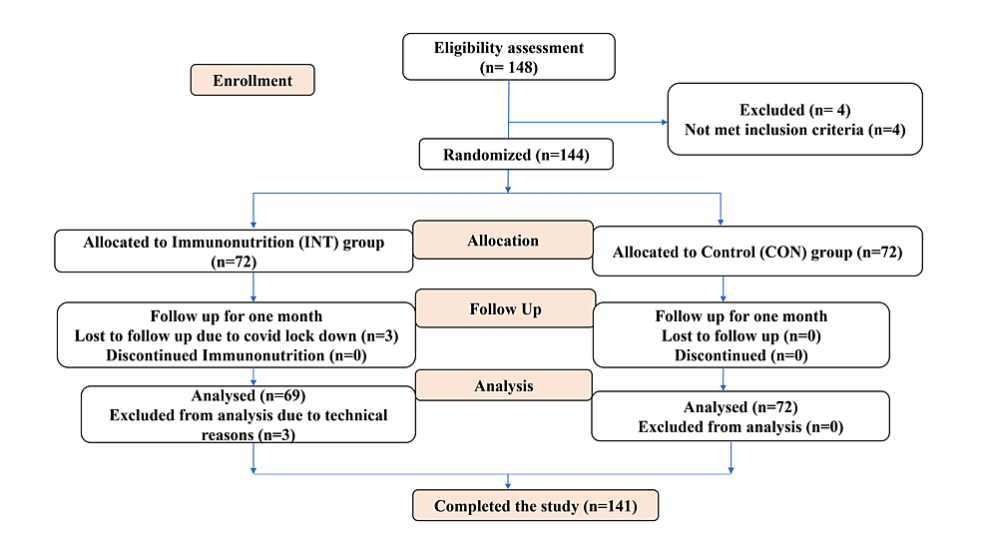
Consolidated Standards of Reporting Trials (CONSORT) diagram. Both groups were given supervised diet advice along with nutritional counseling.

The severity of the liver disease was computed by the Child-Pugh class (CPC) and model for end-stage liver disease-sodium (MELD-Na) score. Usually, the liver function parameters are altered among cirrhotic patients due to the pathogenicity of the liver and metabolic alterations. Hence, we planned to conduct a study on the effect of immunonutrition on liver function parameters among ESLD patients waiting/referred for LT. The liver function parameters and prothrombin time (PT)/international normalized ratio (INR) were assessed for all patients before and after immunonutrient therapy.

Statistical analysis was performed using SPSS for Windows, version 19.0 (SPSS Inc, Chicago, IL, USA). The comparison of the quantitative variables between two groups was conducted using the independent Student’s t-test, and the comparison between more than two groups was performed using a one-way analysis of variance (ANOVA). The means of the difference were estimated using an independent-sample t-test. All statistical analyses were conducted at a 5% level of significance, and a p-value <0.05 was considered significant.

## Results

ESLD patients referred for LT were assessed. The demographic and clinical characteristics of patients in the control (n = 72) and intervention (n = 69) groups are presented in Table 1. The mean age of ESLD patients and the proportion of males were similar in both groups. The majority of patients with liver disease had alcoholism as the major etiology in both groups. The occupation of the patients was mainly agricultural work and other jobs, with a low socioeconomic status. Patients with comorbidities such as diabetes mellitus, hypertension, hypothyroidism, coronary heart disease, or a combination of these were identified. Other clinical factors such as comorbidity, smoking, edema, and food type were not associated with the status of the intervention.

**Table 1:** Baseline demographic and clinical characteristics of ESLD patients in the control and intervention groups. *: statistically significant at p <0.05; #: mean (SD). ESLD: end-stage liver disease

Demographic/Clinical characteristics	Category	Control (n = 72) %	Immunonutrition (n = 69) %	P-value
Age^#^		49.6 (11.4)	49.3 (9.3)	0.85
Height (cm)^#^		166.2 (7.2)	163.3 (8.3)	<0.05*
Gender	Males	60 (83.3)	53 (76.8)	0.33
	Females	12 (16.7)	16 (23.2)	
Occupation	Agriculture	13 (18.1)	19 (27.5)	0.06
	Shop	7 (9.7)	11 (15.9)	
	Driver	7 (9.7)	7 (10.1)	
	Government job	3 (4.2)	3 (4.3)	
	Others	31 (43.1)	13 (18.8)	
	Housewife	11 (15.3)	16 (23.2)	
Marital status	Married	69 (95.8)	69 (100)	0.25
	Unmarried	3 (4.2)	0	
Etiology	Alcoholism	33 (45.8)	39 (56.5)	<0.05*
	Cryptogenic	13 (18.1)	17 (24.6)	
	Hepatitis (B and C)	17 (23.6)	13 (18.8)	
	Others	9 (12.5)	0	
Comorbidity	Yes	17 (23.6)	14 (20.3)	0.74
	No	55 (76.4)	51 (73.9)	
Alcoholism	Yes	36 (50)	39 (56.5)	0.4
	No	36 (50)	30 (43.5)	
Smoking	Yes	6 (8.3)	6 (8.7)	0.9
	No	66 (91.7)	63 (91.3)	
Edema	Yes	43 (59.7)	38 (55.1)	0.58
	No	29 (40.3)	31 (44.9)	
Food type	Vegetarian	4 (5.6)	6 (8.7)	0.53
	Non-vegetarian	68 (94.4)	63 (91.3)	

The comparison of LFT parameters, CPC, and MELD-Na score among ESLD patients during pre and post-therapy in control and immunonutrition groups is presented in Table 2.

**Table 2:** Comparison of LFT parameters, CPC, and MELD-Na scores among ESLD patients in the control and immunonutrition groups at baseline and at one month. #: mean (SD); $: median (IQR). LFT: liver function test; CPC: Child-Pugh class; MELD-Na: model for end-stage liver disease-sodium; ESLD: end-stage liver disease; CON: control; INT: intervention; AST: aspartate aminotransferase; ALT: alanine aminotransferase; ALP: alkaline phosphatase; GGT: gamma-glutamyl transferase; PT: prothrombin time

LFT parameters/MELD-Na score/CPC	Pre-therapy (baseline)			Post-therapy (at one month)		
	Pre-CON (n = 72)	Pre-INT (n = 69)	P-value	Post-CON (n = 72)	Post-INT (n = 68)	P-value
Total protein^#^ (g/dL)	7.35 (1.2)	7.46 (0.8)	0.51	7.13 (0.9)	7.40 (0.8)	0.08
Albumin ^#^ (g/dL)	3.14 (0.9)	3.3 (0.8)	0.26	3.16 (0.9)	3.43 (0.7)	0.06
Total bilirubin^$^ (mg/dL)	1.92 (2.3)	2.11 (2.2)	0.74	1.80 (2.3)	1.9 (2.2)	0.74
Direct bilirubin (mg/dL)	0.59 (1.1)	0.60 (0.9)	0.85	0.57 (0.9)	0.58 (0.8)	0.77
AST^$^ (IU/L)	50 (44.5)	48 (43)	0.63	50.5 (42.5)	53.5 (44.25)	0.89
ALT^$^ (IU/L)	26 (16.75)	30 (22.5)	0.36	28 (20)	29.5 (21)	0.43
ALP^$^ (IU/L)	122 (64.75)	128 (77)	0.34	116 (60)	128.5 (72.0)	0.23
GGT^$^ (IU/L)	44 (49.5)	46 (43.5)	0.38	35 (40.5)	32 (20.5)	0.57
PT^$^ (seconds)	16.5 (5.3)	15.4 (4.5)	0.45	16.5 (4.4)	15.6 (4.1)	0.39
INR^$^	1.3 (0.4)	1.2 (0.3)	0.63	1.3 (0.4)	1.3 (0.3)	0.32
MELD-Na score^$^	17 (10)	14 (9.5)	0.04	15 (10)	15 (9.8)	0.19
Child-Pugh class						
A	23 (32.0)	25 (36.2)	0.86	23 (32.4)	30 (44.1)	0.36
B	35 (48.6)	31 (45.0)		36 (50.7)	28 (41.2)	
C	14 (19.4)	13 (18.8)		12 (16.9)	10 (14.7)	

The mean total protein value in the control and intervention groups at baseline was 7.35 (1.2) and 7.46 (0.8), respectively, and the results indicated no significant difference (p > 0.05) between the two groups. Similarly, the mean total protein value in post-control patients was 7.13 (0.9), and in post-immunonutrition patients after one month was 7.40 (0.8). The results did not show any statistical significance after intervention therapy. However, the total protein was maintained with therapy. The mean albumin values were 3.14 (0.9) and 3.3 (0.8) in the pre-control and pre-immunonutrition groups, respectively, and the results did not show any statistical significance between them. Similarly, the mean albumin value in the post-control and post-immunonutrition patients was 3.16 (0.9) and 3.43 (0.7), respectively. The mean albumin values in both pre-therapy and post-therapy groups did not show any significant change (p > 0.05) after one month of therapy.

The median total bilirubin values in pre-control and pre-immunonutrition groups were 1.92 (2.3) and 2.11 (2.2), respectively, and the results did not show any statistical significance between them. The median total bilirubin values in post-control and post-immunonutrition groups were 1.80 (2.3) and 1.9 (2.2), respectively, and the results depicted no difference between the two groups after one month of therapy. The median direct bilirubin values in pre-control and pre-immunonutrition were 0.59 (1.10) and 0.60 (0.9), respectively. The median direct bilirubin values in post-control and post-immunonutrition were 0.57 (0.9) and 0.58 (0.8), respectively. The results indicated that both groups in pre and post-therapy did not show any statistical significance (p > 0.05) after one month of therapy.

The median aspartate aminotransferase (AST) levels were 50 (44.5) and 48 (43) in the pre-control and pre-immunonutrition groups, respectively. Similarly, the median AST levels were 50.5 (42.5) and 53.5 (44.25) in post-control and post-immunonutrition groups respectively. Both groups did not show any statistical significance (p > 0.05) after one month. The median alanine aminotransferase (ALT) levels were 26 (16.75) and 30 (22.5) in the pre-control and pre-immunonutrition groups, respectively. Similarly, the median ALT levels were 28 (20) and 29.5 (21) in the post-control and post-immunonutrition groups, respectively. Both groups did not show any statistical significance (p > 0.05) after one month.

The median alkaline phosphatase (ALP) values were 122 (64.75) and 128 (77) in the pre-control and pre-immunonutrition groups, respectively. Similarly, the median ALP levels were 116 (60) and 128.5 (72) in the post-control and post-immunonutrition groups, respectively. Both groups did not show any statistical significance (p > 0.05) after one month. The median gamma-glutamyl transferase (GGT) values were 44 (49.5) and 46 (43.5), respectively, in the pre-control and pre-immunonutrition groups. Similarly, the median GGT values were 35 (40.5) and 32 (20.5) in the post-control and post-immunonutrition groups, respectively. Both groups did not show any statistical significance (p > 0.05) after one month.

The median PT/INR values were 16.5 (5.3)/1.3 (0.4) and 15.4 (4.5)/1.2 (0.3) in the pre-control and pre-immunonutrition groups, respectively. Similarly, the median PT/INR values were 16.5 (4.4)/1.3 (0.4) and 15.6 (4.1)/1.3 (0.3) in the post-control and post-immunonutrition groups, respectively. Both groups did not show any statistical significance (p > 0.05) after one month. In addition, the MELD-Na score and CPC did not show any statistically significant difference between the two groups after therapy.

In general, a comparison of liver function test (LFT) parameters among ESLD patients in pre and post-therapy between control and immunonutrition groups did not show any statistically significant difference in the LFT parameters and MELD-Na score between the intervention and control groups both at baseline and at one month.

The comparison of means of the difference in LFT parameters among ESLD patients in pre and post-therapy in control and immunonutrition groups is presented in Table 3.

**Table 3:** Comparison of means of the difference in LFT parameters among ESLD patients in pre and post-therapy in the control and intervention groups. $: median (IQR); *: statistically significant at p <0.05. LFT: liver function test; ESLD: end-stage liver disease; CON: control; INT: intervention; AST: aspartate aminotransferase; ALT: alanine aminotransferase; ALP: alkaline phosphatase; GGT: gamma-glutamyl transferase

Groups/LFT parameters	Control (n = 72)			Immunonutrition (n = 69)		Mean difference p-value
	Pre-CON	Post-CON	Pre-INT		Post-INT	
Total protein (g/dL)	7.35 (1.2)	7.13 (0.9)	7.46 (0.8)		7.40 (0.8)	0.03*
Albumin (g/dL)	3.14 (0.9)	3.16 (0.9)	3.3 (0.8)		3.43 (0.7)	0.12
Total bilirubin^$^ (mg/dL)	1.92 (2.3)	1.80 (2.3)	2.11 (2.2)		1.9 (2.2)	0.97
Direct bilirubin^$^ (mg/dL)	0.59 (1.1)	0.57 (0.9)	0.60 (0.9)		0.58 (0.8)	0.99
AST^$^ (IU/L)	50 (44.5)	50.5 (42.5)	48 (43)		53.5 (44.25)	0.49
ALT^$^ (IU/L)	26 (16.75)	28 (20)	30 (22.5)		29.5 (21)	0.69
ALP^$^ (IU/L)	122 (64.75)	116 (60)	128 (77)		128.5 (72.0)	0.53
GGT^$^ (IU/L)	44 (49.5)	35 (40.5)	46 (43.5)		32 (20.5)	0.024*

The means of mean difference showed that the total protein after immunonutrition therapy was significant compared to the control group (p < 0.05). Similarly, the GGT values showed significance (p < 0.05) with immunonutrition after one month.

## Discussion

A total of 141 patients were analyzed (69 in the INT group and 72 in the CON group) in our study. All baseline clinical and demographic characteristics in the two groups did not show any significant differences. In our study, the majority of patients with the disease were males (83.3% in the CON versus 76.8% in the INT group), having alcoholism as the etiology in both groups (45.8% in the CON group and 56.5% in the INT group). These results were reinforced in a related study, where 61% had alcoholism as the causative factor, and the majority (72%) were males [5].

The comparison of LFT parameters among ESLD patients in pre and post-therapy in control and immunonutrition groups did not show any statistically significant difference in the LFT parameters between the intervention and control groups both at baseline and at one month in our study. These findings were supported by a study among chemotherapy-receiving cancer patients which reported no significant difference in the biochemical test parameters of nutrition such as albumin, pre-albumin, and total lymphocytes [6]. However, most biochemical parameters along with nutritional parameters were maintained within the normal range in both groups even after one month in our ESLD patients, indicating that immunonutrition and supervised diet advice help in maintaining liver function without further deterioration. Another meta-analysis on perioperative supplementation of immunonutrition did not show any major difference in the liver function parameters except AST levels. The low levels of AST values alone indicated that liver function improved with immunonutrition [7]. Serum albumin was considered as a measure of morbidity and mortality instead of nutrition level as the albumin values can be affected by various factors such as inflammatory response and undernutrition [6]. The means of the difference showed that the total protein after immunonutrition therapy showed a significant difference compared to the control group (p < 0.05). Similarly, the GGT values also showed significance (p < 0.05) with immunonutrition after one month of therapy. Two RCTs reported a remarkable reduction in the AST levels in the immunonutrient therapy group against the standard diet group, showing improvement in liver function [8,9]. Other RCTs highlighted that the serum ALT, total bilirubin, and direct bilirubin levels did not have a statistically significant difference between the intervention group and standard diet group [8,9], but showed a decreasing trend of these liver function parameters in the intervention group. Total protein and serum albumin levels were significantly reduced in patients who received immune-enhancing formula compared to baseline in the early postoperative days and showed a trend to normalization afterward [10].

Our study aimed to identify the effect of immunonutrition on liver function parameters between control and intervention groups at baseline and after one month among ESLD patients. Immunonutrition and supervised diet advice did not improve liver function among ESLD patients. The limitations of our study were that we included patients both in CPC B and C and based on our findings a more extensive study needs to be conducted only with CPC B or CPC C. This study helps to develop ideas about the early nutritional plan during the LT waiting period.

## Conclusions

The comparison of LFT parameters in control and immunonutrition groups did not show any statistically significant difference. In addition, immunonutrition did not show any beneficial effect in augmenting the liver function status of ESLD patients. Hence, we strongly recommend early nutritional planning with nutritional counseling among patients with ESLD waiting/referred for LT to improve the clinical outcomes and liver function.
